# Supplementary material for: Patterns of Intron Gain and Loss in Fungi
Source: PLoS Biol. 2004 Nov 30;2(12):e422. doi: 10.1371/journal.pbio.0020422 (PMC532390; doi:10.1371/journal.pbio.0020422)
Supplement: Table S1 — Also available at http://genes.mit.edu/NielsenEtAl/. (4.3 MB ZIP). [file pbio.0020422.st001.zip › NielsenEtAl/html/814.html]

AN4986.1.NCU02176.1.MG02749.1.FG08717.1


```
 CLUSTAL W (1.82) Multiple Sequence Alignments - Introns Inserted


Sequence 1: MG02749.1	296 aa
Sequence 2: FG08717.1	270 aa
Sequence 3: NCU02176.1	252 aa
Sequence 4: AN4986.1	255 aa
Alignment Length: 328 aa
Number Identitical Residues: 89 aa
Alignment Score (without introns) 4571


MG02749.1 	MPKDRKQAGA--KPAARGQSQAHAAAATSQSLSSQQRQQQEAQQPPAWPIFKPTLPVSDL
NCU02176.1	MPKPKKAPATNGKPAHASTPSAAAAATP------------ASSAPPSWPPFKPSLPITDL
FG08717.1 	MPKPKN------KTTKPGKVEVSALPP---------------QAPPSWPVFKPPLPVVEL
AN4986.1  	MPKSKPSK----KDHHPSQSLSH-------------------QQPPNWPPLRPLVPSSDL
          	*** :       *    .                        . ** ** ::* :*  :*

MG02749.1 	SLD-TIVPGKVVVMHNFFPRSLCRDYVAFLKTLPLVTTPARPKKGEAVRQNDRFQVNDPD
NCU02176.1	TFD-TLVDDKVVVLRNFFPKSLCRDYVSFLSTLPLITTPGKPKRGMAVRVNDRFQIDDQN
FG08717.1 	APEPHPLTSKVVLIPSFFPRSLCRDYVAFLKTLPLQTTPGRPKRGEAVRVNDRFQVDSQD
AN4986.1  	YLD-PLLPDQIYLIRNFLPASLCKTYVSFLASLPLTTTPGKPKKGDAVRVNDRFQIQDAT
          	  :   : .:: :: .*:* ***: **:** :*** ***.:**:* *** *****::.  

MG02749.1 	FAARLWLQTGLKEALTA1DC------------KPIPCASR~GGDVVGLSPNIR~VYRYTK
NCU02176.1	FADRLWYETGLKEAVLG~G---------------------~-----GLEH-LW2IYRYTK
FG08717.1 	FATRLWEQTGLKEALLQ~G---------------DVEEKW2GGEPVGLSPNIR~IYRYSK
AN4986.1  	FAENLWRGTALKELVTN~PVSVDDGVGEYSDGGRSMKEIW~GGEPLGLNANIR~VYRYSP
          	** .**  *.*** :     : ... .. :..         ...  **. .:  :***: 

MG02749.1 	GQHFAPHY1DDSNTLTLDPPPTRPQDAGTNSSTISKPPSVAVRTTWTLLLYLTGTAGGDD
NCU02176.1	GQFFDAHY1DDSNKITLTLPPSDDIPS-------PNRQQVPAKTTWTLLLYLTSASEG--
FG08717.1 	GQFFDCHY1DDSNNLTLPLDPPMP-----------------VKTTWTLLLYLTSTAEG--
AN4986.1  	GQFFAQHY1DDSNTLSFLSPSMPPKP---------------ARTTWTLLIYLST------
          	**.*  ** ****.:::   .                    .:******:**:       

MG02749.1 	QCSGGETVFYPNDRVS--RKEEIAVAPQTGLLLLHKHGNDCMLVS------FTPDDLGDA
NCU02176.1	-CIGGETVFYPHDRQV--AKEAIAVAPETGMLLLHKHGNDCMLHE---------GREVTA
FG08717.1 	-CVGGETVFYPRDRRS--LREEIAVPLDTGMLLLHKHGDDCLLVISPLPLSFYHARHGSQ
AN4986.1  	-CTGGETVFYPQRTRSDPNPEPISVAPEMGMALLHRHGDQCLLHE---------GKEVTH
          	 * ********.    ..  * *:*. : *: ***:**::*:*                 

MG02749.1 	T-----ILFAYFSRLTRGWHTARGKRSD----
NCU02176.1	G--------EKWIIRTD-LCVARGGR------
FG08717.1 	NSDHRLTIISSFSMKDEKSLQARNGSSGLIFA
AN4986.1  	G--------EKWVLRSD-LVVAR---------
          	           :         **
```
